# Prescription appropriateness of anti-diabetes drugs in elderly patients hospitalized in a clinical setting: evidence from the REPOSI Register

**DOI:** 10.1007/s11739-023-03254-3

**Published:** 2023-03-25

**Authors:** Elena Succurro, Alessio Novella, Alessandro Nobili, Federica Giofrè, Franco Arturi, Angela Sciacqua, Francesco Andreozzi, Antonello Pietrangelo, Giorgio Sesti, REPOSI Investigators, Francesco Perticone, Francesco Violi, Salvatore Corrao, Alessandra Marengoni, Mauro Tettamanti, Luca Pasina, Carlotta Franchi, Carlotta Franchi, Mauro Tettamanti, Gabriella Miglio, Mauro Tettamanti, Ilaria Ardoino, Silvia Cantiero, Domenico Prisco, Elena Silvestri, Giacomo Emmi, Alessandra Bettiol, Irene Mattioli, Matteo Mazzetti, Gianni Biolo, Michela Zanetti, Giacomo Bartelloni, Michele Zaccari, Massimiliano Chiuch, Ilaria Martini, Matteo Pirro, Graziana Lupattelli, Vanessa Bianconi, Riccardo Alcidi, Alessia Giotta, Massimo R Mannarino, Domenico Girelli, Fabiana Busti, Giacomo Marchi, Mario Barbagallo, Ligia Dominguez, Vincenza Beneduce, Federica Cacioppo, Salvatore Corrao, Giuseppe Natoli, Salvatore Mularo, Massimo Raspanti, Christiano Argano, Federica Cavallaro, Marco Zoli, Giuseppe Orio, Eleonora Magnolfi, Giovanni Serafini, Mattia Brunori, Ilaria Lazzari, Angelo Simili, Giovanna Fabio, Margherita Migone De Amicis, Giacomo Luca, Natalia Scaramellini, Valeria Stefano, Simona Leoni, Sonia Seghezzi, Alessandra Danuto Di Mauro, Diletta Maira, Marta Mancarella, Tiziano Lucchi, Marta Clerici, Simona Leoni, Giulia Bonini, Federica Conti, Silvia Prolo, Maddalena Fabrizi, Miriana Martelengo, Giulia Vigani, Paola Nicolini, Antonio Sabatino, Emanuela Miceli, Martina Pisati, Lavinia Pitotti, Valentina Antoci, Ginevra Cambiè, Lavinia Pitotti, Valentina Antoci, Roberto Pontremoli, Valentina Beccati, Giulia Nobili, Giovanna Leoncini, Jacopo Alberto, Federico Cattaneo, Luigi Anastasio, Lucia Sofia, Maria Carbone, Francesco Cipollone, Ilaria Rossi, Emanuele Valeriani, Damiano D’Ardes, Alessia Cipollone, Lucia Esposito, Simona Sestili, Ermanno Angelucci, Gerardo Mancuso, Daniela Calipari, Mosè Bartone, Roberto Manetti, Marta Sircana, Maria Berria, Alessandro Delitala, Maurizio Muscaritoli, Alessio Molfino, Enrico Petrillo, Antonella Giorgi, Christian Gracin, Giovanni Imbimbo, Giuseppe Romanelli, Alessandra Marengoni, Andrea Volpini, Daniela Lucente, Francesca Manzoni, Annalisa Pirozzi, Alberto Zucchelli, Thelma Geneletti, Antonio Picardi, Giuseppe Bellelli, Maurizio Corsi, Cesare Antonucci, Chiara Sidoli, Giulia Principato, Alessandra Bonfanti, Hajnalka Szabo, Paolo Mazzola, Andrea Piazzoli, Maurizio Corsi, Bruno Tassone, Antonio Brucato Teresa De Falco, Enrica Negro, Martino Brenna, Lucia Trotta, Fabrizio Fabris, Irene Bertozzi, Giulia Bogoni, Tancredi Prandini, Francesco Ratti, Chiara Zurlo, Lorenzo Cerruti, Elisabetta Cosi, Elisa Reni, Roberto Manfredini, Benedetta Boari, Alfredo Giorgi, Ruana Tiseo, Caterina Savriè, Fabio Fabbian, Giuseppe Paolisso, Claudia Catalano, Irene Meo, Carlo Sabbà, Patrizia Suppressa, Giovanni Michele De Vincenzo, Alessio Comitangelo, Emanuele Amoruso, Carlo Custodero, Giuseppe Re, Ivano Barnaba, Andrea Schilardi, Luigi Fenoglio, Andrea Falcetta, Salvatore D’Aniano, Silvia Tiraboschi, Annalisa Cespiati, Giovanna Oberti, Giordano Sigon, Felice Cinque, Lucia Colavolpe, Jaqueline Currà, Francesca Alletto, Natalia Scaramellini, Simona Leoni, Alessandra Danuta Di Mauro, Gianpaolo Benzoni, Flora Peyvandi, Raffaella Rossio, Giulia Colombo, Pasquale Agosti, Erica Pagliaro, Eleonora Semproni, Ciro Canetta, Valter Monzani, Valeria Savojardo, Giuliana Ceriani, Christian Folli, Tiziana Tognin, Francesco Purrello, Antonino Pino, Salvatore Piro, Renzo Rozzini, Lina Falanga, Stefano Boffelli, Camillo Ferrandina, Francesca Mazzeo, Elena Spazzini, Giulia Cono, Giulia Cesaroni, Francesco Violi, Ludovica Perri, Luigina Guasti, Francesca Rotunno, Luana Castiglioni, Andrea Maresca, Alessandro Squizzato, Leonardo Campiotti, Alessandra Grossi, Francesco Dentali, Veronica Behnke, Maria Perticone, Raffaele Maio, Aleandra Scozzafava, Valentino Condoleo, Elvira Clausi, Giuseppe Armentaro, Alberto Panza, Valentino Condoleo, Vincenzo Stanghellini, Eugenio Ruggeri, Sara Vecchio, Ilaria Benzoni, Salvatore Minisola, Luciano Colangelo, Mirella Cilli, Giancarlo Labbadia, Jessica Pepe, Pietro Castellino, Luca Zanoli, Agostino Gaudio, Anastasia Xourafa, Concetta Spichetti, Serena Torre, Alfio Gennaro, Alberto Ballestrero, Fabio Ferrando, Roberta Gonella, Domenico Cerminara, Paolo Setti, Chiara Traversa, Camilla Scarsi, Giuseppe Famularo, Patrizia Tarsitani, Tiziana Morretti, Andrea Aglitti, Stefano Giacco, Davide Firinu, Giulia Costanzo, Salvatore Chessa, Giuseppe Montalto, Anna Licata, Angelo Rizzo, Francesco Corica, Giorgio Basile, Antonino Catalano, Federica Bellone, Concetto Principato, Angelo Cocuzza, Patrizia Mecocci, Carmelinda Ruggiero, Virginia Boccardi, Tiziana Meschi, Andrea Ticinesi, Antonio Nouvenne, Mario Pirisi, Daniele Sola, Mattia Bellan, Roberto Quadri, Erica Larovere, Marco Novelli, Emilio Simeone, Rosa Scurti, Fabio Tolloso, Roberto Tarquini, Alice Valoriani, Silvia Dolenti, Giulia Vannini, Riccardo Volpi, Pietro Bocchi, Alessandro Vignali, Sergio Harari, Chiara Lonati, Federico Napoli, Italia Aiello, Teresa Salvatore, Lucio Monaco, Carmen Ricozzi, Francesca Coviello, Christian Catalini, Alberto Pilotto, Ilaria Indiano, Federica Gandolfo, Davide Gonella, Ranuccio Nuti, Roberto Valenti, Martina Ruvio, Silvia Cappelli, Alberto Palazzuoli, Vittorio Durante, Daniela Tirotta, Giovanna Eusebi, Moreno Tresoldi, Enrica Bozzolo, Sarah Damanti, Massimo Porta, Miriam Gino, Bianca Pari, Edoardo Pace

**Affiliations:** 1 Department of Medical and Surgical Sciences, University Magna Graecia of Catanzaro, Viale Europa, 88100 Catanzaro, Italy; 2 Department of Health Policy, Istituto Di Ricerche Farmacologiche Mario Negri IRCCS, 20156 Milan, Italy; 3 Division of Internal Medicine 2nd Center for Haemochromatosis, University Hospital of Modena, 41124 Modena, Italy; 4 Department of Clinical and Molecular Medicine, University of Rome-Sapienza, 00189 Rome, Italy

**Keywords:** Diabetes, Elderly people, Hospitalized patients, Prescription appropriateness, Anti-diabetes drugs

## Abstract

Diabetes is an increasing global health burden with the highest prevalence (24.0%) observed in elderly people. Older diabetic adults have a greater risk of hospitalization and several geriatric syndromes than older nondiabetic adults. For these conditions, special care is required in prescribing therapies including anti- diabetes drugs. Aim of this study was to evaluate the appropriateness and the adherence to safety recommendations in the prescriptions of glucose-lowering drugs in hospitalized elderly patients with diabetes. Data for this cross-sectional study were obtained from the REgistro POliterapie–Società Italiana Medicina Interna (REPOSI) that collected clinical information on patients aged ≥ 65 years acutely admitted to Italian internal medicine and geriatric non-intensive care units (ICU) from 2010 up to 2019. Prescription appropriateness was assessed according to the 2019 AGS Beers Criteria and anti-diabetes drug data sheets.Among 5349 patients, 1624 (30.3%) had diagnosis of type 2 diabetes. At admission, 37.7% of diabetic patients received treatment with metformin, 37.3% insulin therapy, 16.4% sulfonylureas, and 11.4% glinides. Surprisingly, only 3.1% of diabetic patients were treated with new classes of anti- diabetes drugs. According to prescription criteria, at admission 15.4% of patients treated with metformin and 2.6% with sulfonylureas received inappropriately these treatments. At discharge, the inappropriateness of metformin therapy decreased (10.2%, P < 0.0001). According to Beers criteria, the inappropriate prescriptions of sulfonylureas raised to 29% both at admission and at discharge. This study shows a poor adherence to current guidelines on diabetes management in hospitalized elderly people with a high prevalence of inappropriate use of sulfonylureas according to the Beers criteria.

**Supplementary Information:**

The online version contains supplementary material available at 10.1007/s11739-023-03254-3.

## Introduction

Type 2 diabetes is an increasing global health burden with a global prevalence reaching pandemic proportions. This rising prevalence has been attributed mainly to the ageing of populations [1]. It is estimated that there are currently 537 million people living with diabetes worldwide and among these 135.6 million are individuals aged 65–99 years [1, 2]. Prevalence of type 2 diabetes increases with age with the highest prevalence (24.0%) being observed in individuals aged 75–79 years [1]. Furthermore, the prevalence of type 2 diabetes in hospitalized patients aged 65–75 years and over 80 years of age has been estimated to be 20 and 40%, respectively [3–6]. It is estimated that the number of people with diabetes will continue to rise rapidly in the next years. Indeed, future projections of International Diabetes Federation (IDF) Diabetes Atlas suggest that by 2045 the absolute number of people with type 2 diabetes will have increased by 46% and the number of people older than 65 years with diabetes will reach 195.2 million by 2030 and 276.2 million by 2045 [1, 2].

Older adults with type 2 diabetes have higher rates of coexisting illnesses, such as hypertension, coronary heart disease, stroke, and functional disability, than those without diabetes [7, 8]. Furthermore, older adults are more apt to require hospitalization than younger adults, and, particularly, those with diabetes are at very high risk of hospitalization. Additionally, older adults with type 2 diabetes are also at greater risk than older nondiabetic adults for several common geriatric syndromes, such as cognitive impairment, injurious falls, polypharmacy, increasing the risk of drug side effects, and drug-to-drug interactions [7–9]. For these conditions, special care is required in prescribing and monitoring pharmacologic therapies including anti-diabetes drugs, in older adults [9, 10].

Insulin therapy is the preferred pharmacological approach to manage hyperglycemia in hospitalized patients with type 2 diabetes [3]. For patients in non-intensive care units (ICU) settings, subcutaneous basal insulin alone or in combination with prandial insulin, is effective and safe [3]. Selecting the treatment regimen in elderly patients is based on patient’s nutritional status, body weight, and hypoglycemia risk. The use of noninsulin antihyperglycemic agents is not recommended for the management of hyperglycemia in hospitalized patients with type 2 diabetes [3].

Metformin is considered the first-line therapy for older adults with type 2 diabetes due to its efficacy and safety profile [8–11]. However, metformin should be temporarily discontinued during hospitalizations, before procedures, and when acute illness may compromise renal or liver function or may induce heart failure because of the increased risk of lactic acidosis [8, 10].

Sulfonylureas are associated with increased risk of hypoglycemia and should be used with caution in older people [8]. Notably, the American Geriatrics Society (AGS) Beers Criteria 2019 recommended to avoid glimepiride and glibenclamide for the high risk of severe prolonged hypoglycemia [12]. The use of thiazolidinediones may precipitate or worsen heart failure and peripheral edema [3].

Instead, there is a particular interest in the use of dipeptidyl peptidase 4 (DPP-4) inhibitors in hospitalized patients with type 2 diabetes for their few side effects and neutral effects on major adverse cardiovascular outcomes [3, 5, 11–15]. Moreover, in hospitalized patients, treatment with DPP-4 inhibitors has been associated with similar glycemic control, and lower rates of hypoglycemia compared with insulin regimens [3, 16, 17]. Nevertheless, it has been reported that saxagliptin treatment is associated with an increased risk of hospitalizations for heart failure, also in elderly and very elderly patients [18]. The cardiovascular (CV) safety data on the effects of DPP-4 are conflicting since some randomized clinical trials and some real-life studies have reported an increased risk of hospitalizations for heart failure [19], while a recent meta-analysis shows that DPP-4 inhibitors do not increase the risk of heart failure [20]. Therefore, the choice of treatment with DPP-4 inhibitors in the elderly patient with type 2 diabetes should take into account of comorbidities, especially heart failure.

Results of cardiovascular outcome trials (CVOT) have shown that treatment with sodium–glucose cotransporter 2 inhibitors (SGLT2i) and GLP-1 receptor agonists (GLP-1 RA) is associated with cardiovascular protection in diabetic patients with established atherosclerotic cardiovascular disease (ASCVD) and in those with higher ASCVD risk with benefits observed also in patients older than 65 years of age [21–30]. However, the increased risk of urinary and genital tract infections observed in patients treated with SGLT2i, the possible occurrence of volume depletion, and the development of diabetic ketoacidosis among patients with type 2 diabetes make the use of SGLT2 inhibitors less attractive in acutely ill hospitalized patients with hyperglycemia [3]. On the other hand, treatment with GLP-1 RA may not be advisable in some frail older patients, particularly those suffering from malnutrition sarcopenia, and cachexia, given that their use is associated with gastrointestinal side effects [3, 9].

The inappropriate use of anti-diabetes drugs is frequent, especially in the elderly hospitalized patients. However, although prior studies have shown a high prevalence of potentially inappropriate prescribing for adults living with type 2 diabetes, none of these studies have used an explicit tool specifically designed to identify inappropriate prescribing among people with diabetes, especially in older people [31].

The aim of this study was to evaluate the appropriateness and the adherence to safety recommendations in the prescriptions of anti-diabetes drugs both at hospital admission and at discharge in a cohort of elderly patients with type 2 diabetes hospitalized in internal medicine and geriatric non-ICU participating in the REPOSI registry study.

## Methods

### Setting

Data for this cross-sectional study were obtained from the register REgistro POliterapie – Società Italiana Medicina Interna (REPOSI), an ongoing collaboration between the Italian Society of Internal Medicine (SIMI), IRCCS Fondazione Ca` Granda Ospedale Maggiore Policlinico, and the Istituto di Ricerche Farmacologiche Mario Negri IRCCS. The REPOSI is a multicenter and prospective register that started in 2008 in order to collect clinical and therapeutic information on patients aged 65 years or older acutely admitted to 102 Italian internal medicine and geriatric non-ICU during four index weeks during each season. Data collections were continued in 2010, 2012, 2014, 2016 and 2019.

The project’s design has been previously described in detail [32–34]. Briefly, patients were eligible for REPOSI if: (1) they were admitted to one of the participating regional internal medicine non-ICU during the four index weeks chosen for recruitment (one in February, one in June, one in September, and one in December); (2) their age was 65 years or older; (3) they gave informed consent. Each non-ICU had to enroll at least five consecutive eligible patients during each index week, recording data on socio-demographic details, diagnoses, treatment (including all drugs taken at hospital admission, and those recommended at discharge). Then, a final database was created and checked by the Istituto di Ricerche Farmacologiche Mario Negri IRCCS. All patients with and without diabetes were included in the present study analysis. Participation was voluntary, and all patients provided signed informed consent. REPOSI was approved by the Ethics Committee of the participating centers. The study was conducted according to Good Clinical Practice and the Declaration of Helsinki.

### Data collection

**Fig. 1:**
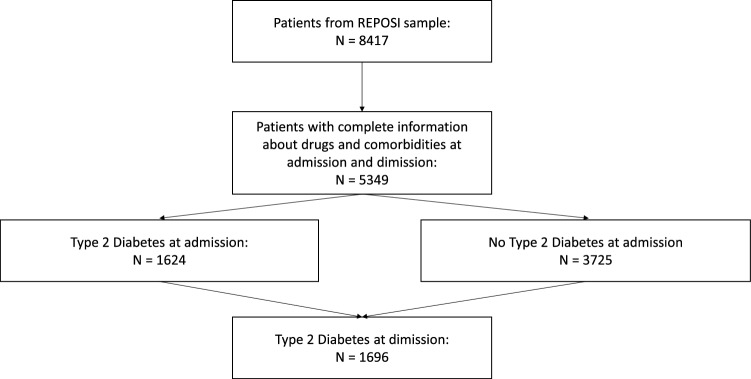
Flowchart of the study population

REPOSI register includes 8417 older adults admitted to the participating internal medicine and geriatric wards enrolled from 2010 up to 2019. For this study, data from 5349 patients with complete information were evaluated (Fig. 1). According to the ADA criteria [35], individuals were classified as having type 2 diabetes when fasting plasma glucose was ≥ 126 mg/dl (> 7 mmol/l), or were treated with antidiabetic drugs. Patients with type 1 diabetes were excluded from enrollment from participating centers. All patients with type 2 diabetes were screened in order to determine what type of anti-diabetes drugs they were prescribed, both at hospital admission and discharge. Hospital admission therapy refers to the treatment taken at home before the admission*.* Anti-diabetes drugs use at admission and discharge was coded according to the Anatomic Therapeutic Chemical (ATC) Classification System. We used the following ATC codes: insulin therapy: A10A, metformin: A10BA, Sulfonylureas: A10BB, Glinides: A10BX02, Pioglitazone: A10BG03, DPP-4 inhibitors: A10BH, GLP-1 RA: A10BJ, SGLT2 inhibitors: A10BK, Acarbose: A10BF01.

### Socio-demographic and clinical characteristics

Socio-demographic variables, such as age class, marital status, living arrangement, and need for assistance in daily living, were considered, along with laboratory findings in patients with diabetes compared to the ones without it. The following clinical characteristics were evaluated: cognitive status (assessed by the Short-Blessed-Test (SBT) [36]; performance in activities of daily living at hospital admission (measured by means of the Barthel Index (BI) [37]; severity and comorbidity index (assessed by the Cumulative-Illness-Rating-Scale (CIRS-s and CIRS-c, respectively)) [38]; glomerular filtration rate (eGFR) (using the Chronic Kidney Disease Epidemiology Collaboration formula) [39]; length of hospital stay; drug prescriptions (at admission and at discharge). Polypharmacy was defined by the contemporary chronic use of 5 or more drugs [40].

### Criteria for inappropriate/appropriate prescription and adherence to guidelines recommendations

Prescription appropriateness was assessed according to the 2019 American Geriatrics Society Beers Criteria [12], and the indications according to the European Medicines Agency (EMA) and Italian Medicines Agency (AIFA) anti-diabetes drug data sheets. Briefly, in patients with type 2 diabetes hospitalized for exacerbations of chronic diseases or in the case of acute diseases, it is recommended to prescribe insulin therapy [8, 12]. Metformin therapy is inappropriate for eGFR values < 30 mL/min/1.73m^2^, during acute illness, acute myocardial infarction, metabolic acidosis, shock and respiratory failure for the increased risk of lactic acidosis [8, 12]. Sulfonylureas and other insulin secretagogues are not recommended in older adults for the increased risk of hypoglycemia [8, 12]. Furthermore, sulfonylureas are inappropriate during severe kidney and liver failure and acute illness [8, 12]. Pioglitazone is inappropriate during heart failure, several liver impairment and bladder cancer [8, 12]. Moreover, it is recommended caution in the use of pioglitazone in case of osteoporosis and history of bone fractures [8, 12]. GLP-1 RA therapy is inappropriate in case of acute pancreatitis and end-stage renal disease [8, 12]. SGLT2 inhibitors are inappropriate during severe renal failure [8, 12].

### Statistical analysis

We divided our sample in two groups according to the presence of type 2 diabetes at admission in hospital. For each patient the presence of this condition was defined using directly the diagnosis and/or the prescriptions of anti-diabetes drugs. The patients' socio-demographic characteristics were presented using standard descriptive statistics. We tabulated percentages for discrete variables, mean and standard deviations for continuous variables. Differences between the two groups were evaluated with Pearson’s chi-squared test. Mean and standard deviations for numerical variables were evaluated with Mann Whitney’s test. Normality for clinical continuous features was checked with Kolmogorov–Smirnov and Anderson–Darling tests.

Successively, on the subgroup of all diabetic subjects regardless of whether it occurred before or during the hospitalization, we performed a pre-post analysis using McNemar’s test in order to evaluate the change of anti-diabetes prescription from admission to discharge. Analogue analyses were performed to assess the appropriateness of each anti-diabetes classes investigated.

Successively, on the sample of diabetic subjects, we studied the relationship between mortality at 3 months after discharge and appropriateness of the antidiabetic therapy according to the combination of the EMA and AIFA data sheets and 2019 AGS Beers criteria; we conducted a logistic model regression first univariately and then adjusting Odds Ratios (OR) for age, sex and comorbidity index. A logistic regression analysis adjusted by age, gender, number of drugs, comorbidity index and eGFR (dichotomized using a threshold of 30 mL/min/1.73m^2^, according to prescriptive criteria) was conducted to evaluate causes of inappropriateness in prescriptions of anti-diabetic drugs. Confidence Intervals (CI) were calculated using Wald’s test.

For each statistical test, the significance criterion (alpha) was set at 0.05.

All analyses were performed using SAS software, version 9.4 (SAS Institute, Inc.; Cary, NC).

## Results

### Clinical characteristics of the elderly population according to diabetes diagnosis

For this analysis, 5349 patients acutely admitted to 102 Italian internal medicine and geriatric non-ICU during the period from 2010 up to 2019 were evaluated; among them, 1624 (30.3%) had diagnosis of type 2 diabetes, and 3725 were patients without history of diabetes (69.7%) (Fig. 1). During the hospitalization 72 patients were diagnosed as having newly diagnosed type 2 diabetes leading to a total number of 1696 patients with diagnosis of diabetes at hospital discharge. All clinical parameters evaluated with Kolmogorov–Smirnov and Anderson–Darling tests resulted not normally distributed (all p < 0.01 using the first test and all p < 0.005 using the second one).

As shown in Table 1, patients with type 2 diabetes were more likely to be men, younger, married, not living alone, and ex-smoker as compared with nondiabetic patients (Table 1).

**Table 1:** Socio-demographic and anthropometrics characteristics of the elderly population according to the presence of diabetes

	Type 2 Diabetes (n = 1696)	No Type 2 Diabetes (n = 3653)	P value
Gender, n (%)			
Female	800 (46.8)	1978 (54.2)	< 0.0001
Male	895 (53.2)	1674 (45.8)	
Missing	1	1	
Age (yrs), mean ± SD	78.4 ± 7.0	80.0 ± 7.7	< 0.0001
Civil Status, n (%)			
Married, *n* (*%*)	922 (56.7)	1825 (51.9)	0.0015
Widow, *n* (*%*)	549 (33.7)	1351 (38.4)	0.0012
Separated, *n* (*%*)	29 (1.8)	51 (1.5)	0.37
Divorced, *n* (*%*)	27 (1.7)	54 (1.5)	0.74
Single, *n* (*%*)	100 (6.1)	234 (6.7)	0.49
Missing	69	138	
*Live with, n (%)*			
Living alone	324 (20.3)	897 (25.9)	< 0.0001
With Partner	770 (48.2)	1546 (44.6)	0.0197
With Children	254 (15.9)	527 (15.2)	0.54
With Partner & Children	122 (7.6)	198 (5.7)	0.0094
Other	129 (8.1)	295 (8.5)	0.59
Missing	97	190	
Having a caregiver, *n* (*%*)	871 (52.1)	1843 (51.2)	0.54
Missing	25	55	
*Alcohol, n* (*%*)			
Never,	929 (57.4)	2018 (57.3)	0.99
Ex-drinker	171 (10.6)	391 (11.1)	0.56
Drinker	230 (14.2)	465 (13.2)	0.34
Social Drinker,	290 (17.9)	646 (18.4)	0.70
Missing	76	133	
*Smoking status, n* (*%*)			
Never smoked	828 (50.8)	2017 (56.8)	< 0.0001
Ex-Smoker	658 (40.4)	1217 (34.3)	< 0.0001
Smoker	144 (8.8)	317 (8.9)	0.91
Missing	66	102	
BMI (*kg/m*^*2*^), mean ± SD	27.8 ± 5.5	25.2 ± 4.7	< 0.0001
*BMI classes, n (%)*			
BMI < 18.5	17 (1.2)	162 (5.1)	< 0.0001
BMI ≥ 18.5 and < 24.9	483 (33.0)	1481 (46.9)	< 0.0001
BMI ≥ 25 and < 29.9	574 (39.2)	1075 (34.1)	0.0007
BMI ≥ 30	389 (26.6)	437 (13.9)	< 0.0001
Missing	233	498	< 0.0001
*Comorbidities, n* (*%*)			
Hypertension	1038 (61.2)	1897 (51.9)	< 0.0001
Myocardial Infarction	99 (5.8)	153 (4.2)	0.0081
Peripheral Vascular Disease	306 (18.0)	521 (14.3)	0.0004
Cerebrovascular Disease	403 (23.8)	925 (25.3)	0.22
Heart failure	587 (34.6)	1045 (28.6)	< 0.0001
COPD	447 (26.4)	956 (26.2)	0.89
Rheumatic disease	63 (3.7)	162 (4.4)	0.22
Liver disease	240 (14.2)	355 (9.7)	< 0.0001
Dementia	157 (9.3)	467 (12.8)	0.0002
Chronic Kidney Disease	701 (41.3)	1003 (27.5)	< 0.0001
Cancer	87 (5.1)	227 (6.2)	0.11
Previous hospitalization, *n (%)*	775 (45.7)	1651 (45.2)	0.73
Institutionalized, *n (%)*	95 (5.6)	178 (4.9)	0.26
Missing	12	30	
Length of hospital stay (*days*), mean ± SD	12.7 ± 13.7	12.0 ± 10.4	0.07
Missing	20	29	

Data are reported as mean ± SD, unless otherwise indicated. BMI = body mass index

Patients with type 2 diabetes had higher BMI (27.8 ± 5.5 kg/m^2^ vs 25.2 ± 4.7 kg/m^2^, P < 0.0001), and were more often overweight (39.2% vs 34.1%, P = 0.0007) and obese (26.6% vs 13.9%, P < 0.0001) than nondiabetic patients (Table 1). Moreover, a significant higher proportion of patients with type 2 diabetes had comorbidities, such as hypertension, myocardial infarction, peripheral vascular disease, heart failure, liver disease, and chronic kidney disease with significant higher creatinine levels and lower eGFR as compared with nondiabetic patients (Table 1, Table 2). Furthermore, even if we observed a higher proportion of dementia in nondiabetic individuals than patients with type 2 diabetes, no significant differences were observed regarding overt cognitive impairment between patients with and without diabetes (Table 1, Table 2).

**Table 2:** Clinical and laboratory characteristics of the elderly population according to the presence of diabetes

	Type 2 Diabetes (n = 1696)	No Type 2 Diabetes (n = 3653)	P value
Systolic blood pressure (*mmHg*), mean ± SD	134.3 ± 22.1	131.6 ± 22.0	< 0.0001
Missing	8	28	
Diastolic blood pressure (*mmHg*), mean ± SD	73.6 ± 12.2	73.7 ± 16.2	0.71
Missing	7	24	
Heart rate (*bpm*), mean ± SD	79.3 ± 16.0	79.9 ± 17.0	0.20
Missing	11	39	
Fasting Glucose *(mgl/dL*, mean ± SD	160.0 ± 83.6	108.5 ± 31.7	< 0.0001
Missing	51	154	
Total cholesterol (*mg/dl*), mean ± SD	150.0 ± 43.4	158.3 ± 45.9	< 0.0001
Missing	471	1104	
Creatinine (*mg/dl*), mean ± SD	1.4 ± 0.9	1.2 ± 0.8	< 0.0001
Missing	20	52	
eGFR (mL/min/1.73m^2^), mean ± SD	55.9 ± 24.5	61.3 ± 23.5	< 0.0001
*eGFR class, n (%)*			
eGFR class I K-DOQI	64 (3.8)	101 (2.8)	0.0488
eGFR class II K-DOQI	231 (13.8)	302 (8.4)	< 0.0001
eGFR class III K-DOQI	647 (38.6)	1273 (35.3)	0.0222
eGFR class IV K-DOQI	592 (35.3)	1569 (43.6)	< 0.0001
eGFR class V K-DOQI	142 (8.5)	356 (9.9)	0.10
Missing	20	52	
Barthel index score, mean ± SD	74.3 ± 29.1	74.4 ± 30.1	0.90
Clinically significant disability (Barthel index ≤ 40), n (%)	238 (15.3)	597 (16.8)	0.18
Missing	70	180	
Short Blessed Test score, mean ± SD	8.7 ± 7.6	8.6 ± 7.8	0.67
Overt Cognitive impairment (SBT ≥ 10), n (%)	605 (41.7)	1338 (40.4)	0.39
Missing	174	413	
Severity index (by CIRS), mean ± SD	1.8 ± 0.3	1.6 ± 0.3	< 0.0001
Severity index (by CIRS)-Excluded diabetes, mean ± SD	1.7 ± 0.3	1.6 ± 0.3	< 0.0001
Comorbidity index (by CIRS), mean ± SD	3.7 ± 1.9	2.7 ± 1.8	< 0.0001
Comorbidity index (by CIRS)-Excluded diabetes, mean ± SD	3.0 ± 1.8	2.7 ± 1.8	< 0.0001
Polypharmacy, n (%)	1342 (79.1)	2000 (54.8)	< 0.0001
Excessive (More than 10 Drugs) n (%)	378 (22.3)	209 (5.7)	< 0.0001
Polypharmacy (excluded drugs for diabetes), n (%)	1135 (66.9)	1997 (54.7)	< 0.0001
Excessive (More than 10 Drugs), n (%)	181 (10.7)	208 (5.7)	< 0.0001
Drug Number, mean ± SD	7.1 ± 3.0	5.1 ± 2.6	< 0.0001
Drug number (excluded drugs for diabetes), mean ± SD	5.9 ± 2.9	5.1 ± 2.6	< 0.0001

Data are reported as mean ± SD, unless otherwise indicated. *CIRS* Cumulative-Illness-Rating-Scale

As expected, patients with type 2 diabetes showed significant higher fasting plasma glucose levels than those without diabetes (Table 2). Moreover, patients with type 2 diabetes exhibited significantly higher levels of systolic blood pressure, and lower levels of heart rate and total cholesterol than nondiabetic individuals (Table 2).

Patients with type 2 diabetes exhibited higher severity index assessed by CIRS-s (1.8 ± 0.3 vs 1.6 ± 0.3, P < 0.0001) and comorbidity index assessed by CIRS-c (3.8 ± 1.9 vs 2.9 ± 1.9, P < 0.0001) as compared with nondiabetic patients, also excluding diabetes in the assessment of CIRS (Table 2). Furthermore, a significant higher proportion of patients with type 2 diabetes took more of 5 chronic drugs and more of 10 chronic drugs (excluded drugs for diabetes) than nondiabetic individuals (Table 2).

### Anti-diabetes therapy in patients with diabetes

At hospital admission, 247 patients among those with diabetes (15.2%) did not receive any type of anti-diabetes therapy, 695 (42.8%) patients were treated with only one drug, 578 (35.6%) received two, while the remaining had the prescription of three or more anti-diabetes drugs in combination.

In particular, 37.7% of patients with type 2 diabetes were treated with metformin, 37.3% with insulin therapy, 16.4% with sulfonylureas, and 11.4% with glinides. Moreover, 2.5% of patients were treated with acarbose and 1.4% with pioglitazone (Table 3). Surprisingly, at admission only 2.8% of patients with type 2 diabetes were treated with DPP-4 inhibitors, 0.1% with GLP1-RA and 0.2% with SGLT2 inhibitors (Table 3).

**Table 3:** Antidiabetic therapy in elderly patients with diabetes

	At admission (n = 1624)	At discharge (n = 1696)	P value
Metformin			
Pure, A10BA02	511 (31.5)	416 (24.5)	< 0.0001
Combinations Included	612 (37.7)	472 (27.8)	< 0.0001
Sulfonylureas			
Pure, A100BB	190 (11.7)	115 (6.8)	< 0.0001
Combinations Included	267 (16.4)	152 (9.0)	< 0.0001
Repaglinide, A10BX02	185 (11.4)	174 (10.3)	0.42
Pioglitazone			
Pure, A10BG03	13 (0.8)	3 (0.2)	0.0016
Combinations Included	23 (1.4)	7 (0.4)	< 0.0001
DPP-IV inhibitors			
Pure, A10BH Combinations Included	28 (1.7)	31 (1.8)	0.44
DPP-IV inhibitors	45 (2.8)	46 (2.7)	0.85
GLP-1 RA			
Pure, A10BJ Combinations Included	2 (0.1)	6 (0.4)	0.05
GLP-1 RA	2 (0.1)	6 (0.4)	0.05
SGLT2 inhibitors			
Pure, A10BK Combinations Included	2 (0.1)	2 (0.1)	1.00
SGLT2 inhibitors	3 (0.2)	4 (0.2)	0.31
Acarbose, A10BF01	40 (2.5)	24 (1.5)	0.0018
Insulin therapy, A10A	605 (37.3)	759 (44.8)	< 0.0001

At hospital discharge, we found a significant decrease in the prescription of metformin (37.7% vs 27.8%, P < 0.0001), sulfonylureas (16.4% vs 9%, P < 0.0001), and pioglitazone (1.4% vs 0.4%, P < 0.0001) and a significant increase in the prescription of insulin therapy (37.7% vs 44.8%, P < 0.0001). Furthermore, at hospital discharge, we observed a nominally significant increase in the prescription of GLP-1 RA (0.1% vs 0.4%, P = 0.05), whereas no differences were observed in prescriptions of DPP-4 inhibitors, SGLT2 inhibitors, and glinides (Table 3).

We therefore analyzed the prescriptions of glucose-lowering drugs stratified by years of enrollment of patients in the REPOSI register, and we did not find significant differences compared with the overall prevalence, although we observed a trend towards a reduction in the prevalence of the prescriptions of sulfonylureas and repaglinide and an increase in those of DPP-IV inhibitors, and slightly of GLP-RA and SGLT2 inhibitors, in the years 2018–2019 compared to the years 2010–2011 (see supplemental materials, Table S1). Furthermore, we analysed the prescriptions of glucose-lowering drugs stratified by geographic areas of centers participating to REPOSI register that enrolled the patients (Northern, Central and Southern Italy). We found a significant lower rate of prescription of sulfonylureas (P = 0.0007) and a greater use of insulin therapy (P = 0.0088) both at admission and at discharge in Southern Italy as compared Northern and Central Italy (see supplemental materials, Table S2).

### Appropriateness of anti-diabetes drugs, at hospital admission and discharge

According to the EMA and AIFA data sheets, among diabetic patients treated with at least one anti-diabetes drug, 99 (7.2%) resulted inappropriately treated at admission. This proportion was reduced at hospital discharge around a half (50 subjects, 3.7%, P < 0.0001). When we also considered the 2019 AGS Beers Criteria, the number of subjects not appropriately treated raised to 284 (20.6%) at admission, with a significative decrement at discharge to 239 units (17.7%, P < 0.049).

At hospital admission, 15.4% of patients treated with metformin, 2.6% treated with sulfonylureas, and 1.1% treated with repaglinide received these treatments inappropriately according to the EMA and AIFA data sheets (Table 4). At hospital discharge, it was observed a decrease in the inappropriateness of metformin therapy (10.2%, P < 0.0001). When we considered the appropriateness of anti-diabetes drugs according to the Beers Criteria, the proportion of not appropriate prescriptions of sulfonylureas raised to 28.5% at hospital admission and was similar being 29% at discharge (P = 0.92) (Table 5).

**Table 4:** Appropriateness of antidiabetic drugs, at hospital admission and discharge

	At admission	At discharge	P value
*Appropriate*			
Metformin Sulfonylureas Thiazolidinediones DPP-IV inhibitors	518 (84.6)	424 (89.8)	< 0.0001
GLP-1 RA	260 (97.4)	151 (99.3)	–
SGLT2 inhibitors Insulin therapy Repaglinide	24 (100)	8 (100)	–
Acarbose	45 (100)	46 (100)	0.014
*Appropriate*	2 (100)	4 (66.7)	0.05
Metformin Sulfonylureas Thiazolidinediones DPP-IV inhibitors	3 (100)	3 (100)	–
GLP-1 RA	605 (100)	759 (100)	–
SGLT2 inhibitors Insulin therapy Repaglinide	183 (98.9)	174 (100)	0.16
Acarbose	40 (100)	24 (100)	–
*Not Appropriate*			
Metformin	94 (15.4)	48 (10.2)	< 0.0001
Sulfonylureas	7 (2.6)	1 (0.7)	0.014
Thiazolidinediones	0 (0)	0 (0)	–
DPP-IV inhibitors	0 (0)	0 (0)	–
GLP-1 RA	0 (0)	2 (33.3)	0.05
SGLT2 inhibitors	0 (0)	0 (0)	–
Insulin therapy	0 (0)	0 (0)	–
Repaglinide	2 (1.1)	(0)	0.16
Acarbose	0 (0)	(0)	–

**Table 5:** Appropriateness of antidiabetic drugs according to also BEERS criteria, at hospital admission and discharge

	At admission	At discharge	P value
*Appropriate*			
Metformin	518 (84.6)	424 (89.8)	< 0.0001
Sulfonylureas	191 (71.5)	108 (71.0)	0.92
Thiazolidinediones	24 (100)	8 (100)	–
DPP-IV inhibitors	45 (100)	46 (100)	–
GLP-1 RA	2 (100)	4 (66.7)	0.05
SGLT2 inhibitors	3 (100)	3 (100)	–
Insulin therapy	486 (80.3)	610 (80.4)	0.99
Repaglinide	183 (98.9)	174 (100)	0.16
Acarbose	40 (100)	24 (100)	–
*Not Appropriate*			
Metformin	94 (15.4)	48 (10.2)	<0.0001
Sulfonylureas	76 (28.5)	44 (29.0)	0.92
Thiazolidinediones	0 (0)	0 (0)	**–**
DPP-IV inhibitors	0 (0)	0 (0)	–
GLP-1 RA	0 (0)	2 (33.3)	0.05
SGLT2 inhibitors	0 (0)	0 (0)	–
Insulin therapy sliding scale	119 (19.7)	149 (19.6)	0.99
Repaglinide	2 (1.1)	0 (0)	0.16
Acarbose	0 (0)	0 (0)	–

Furthermore, we analysed the appropriateness of prescriptions of glucose-lowering drugs stratified by geographic areas of centers participating to REPOSI register and we didn’t observe significant differences between Northern, Central and Southern Italy (see supplemental materials, Table S2).

At hospital admission, the most prevalent cause of inappropriateness among metformin prescriptions was the low levels of eGFR (< 30 mL/min/1.73m^2^) observed in 51 patients (54.3%) while, at discharge, the most prevalent cause was the acute myocardial infarction suffered by 19 patients (39.6%, see supplemental materials, Table S3).

In a logistic regression model adjusted by age, sex, number of drugs, comorbidity index and eGFR (dichotomized using a threshold of 30 mL/min/1.73m^2^, according to the appropriateness prescriptive criteria), only eGFR was significantly associated with inappropriate prescriptions. Notably, patients with eGFR < 30 mL/min/1.73m^2^ exhibited an increased risk of not appropriate treatment compared to patients with higher level of eGFR (OR 2.56 (CI: 1.88–3.49, P < 0.0001).

### Appropriateness of anti-diabetes drugs and outcomes during hospitalization and after discharge

Finally, we have investigated the impact of appropriateness of anti-diabetes drugs according to the combination of the EMA and AIFA data sheets and 2019 AGS Beers criteria on length of hospitalization and mortality at 3 months of subjects with diagnosis of type-2 diabetes. We observed a similar length of hospital stay between appropriated and not appropriated treated patients with type 2 diabetes (12.6 vs 13.1 days, respectively; P = 0.43). Furthermore, we found a higher incidence of mortality at 3 months post-discharge in patients with type 2 diabetes non-appropriately treated as compared to those appropriately treated (8.4% vs 4.7%, P = 0.0196). Notably, patients with type 2 diabetes not appropriately treated exhibited a 1.84-fold increased risk of mortality at 3 months as compared to patients appropriately treated (95% CI 1.09–3.08, P = 0.0215). This increased risk remained statistically significant also in adjusted model including age, sex and comorbidity index (CIRS) (P = 0.0169). In particular, an increased risk of mortality at 3 months after discharge was associated to a point-increment of age (OR = 1.08, CI 1.04–1.11, p < 0.0001), CIRS (OR = 1.12, CI 1.01–1.24, p = 0.031) and men (OR = 1.90, CI 1.20–3.00, p = 0.0058 compared to women).

## Discussion

This study aimed to evaluate the prescribing appropriateness to safety recommendations of anti-diabetes drugs in hospitalized elderly patients with type 2 diabetes both at admission and at discharge. Although previous studies have shown a high prevalence of inappropriate prescribing for outpatients with diabetes, none of them evaluated the prescriptive appropriateness of anti-diabetes drugs in hospitalized elderly patients [31]. The present analysis was performed using data obtained from the database REPOSI, including 5349 patients aged ≥ 65 acutely admitted to 102 Italian internal medicine and geriatric non-ICU wards [32–34]. We found that at hospital admission 16.4% of patients with type 2 diabetes were treated with sulfonylureas. According to the 2019 AGS Beers Criteria, 28.5% of these prescriptions were inappropriate on the basis of the recommendation to avoid the prescription of glimepiride and glibenclamide in elderly people for the high risk of severe prolonged hypoglycemia [12]. On the other hand, the ADA Standards of Care recommends avoiding only the prescription of glibenclamide in elderly people, although the sulfonylureas and other insulin secretagogues with caution for their increased risk of hypoglicemia [8]. Remarkably, although at discharge it was observed a nominal reduction in the prescription of sulfonylureas, 29% of patients with diabetes still remained inappropriately treated with this class of anti-diabetes drugs.

At hospital admission, more than a third of patients with diabetes were treated with metformin, and in 15.4% of the prescriptions were inappropriate according to the EMA and AIFA data sheets. Treatment with metformin is inappropriate in patients with chronic kidney failure and respiratory insufficiency, and during acute illness due to the increased risk of lactic acidosis [8, 12]. We found that hospitalized individuals with diabetes showed a significant impairment of renal function as compared with nondiabetic subjects. In particular, about 40% of patients with diabetes exhibited a moderate or severe impairment in renal function, two conditions in which treatment with metformin is inappropriate.

Furthermore, we observed that individuals with type 2 diabetes exhibited a significant higher severity index and an increase of comorbidities, such as hypertension, myocardial infarction, peripheral vascular disease, heart failure, liver disease, and chronic kidney disease as compared with patients without diabetes. Notably, most of these conditions met the criteria of inappropriateness for treatment with metformin, pioglitazone and other anti-diabetes drugs. The present analysis shows that at hospital admission the most prevalent cause of inappropriateness among metformin prescriptions was the low levels of eGFR observed in 54.3% of the patients, while, at hospital discharge, the most prevalent reason of inappropriateness was the acute myocardial infarction suffered by 39.6% of the subjects. Notably, patients with eGFR < 30 mL/min/1.73m^2^ exhibited an increased risk of not appropriate treatment compared to patients with higher level of eGFR. Therefore, our data underline the critical role of renal function in the evaluation of appropriate antidiabetic treatment in elderly patients hospitalized. Furthermore, respiratory failure and acute illness were the two more common causes of inappropriateness for metformin treatment in patients both at admission (20.2% and 13.8%, respectively) and at discharge (25% and 22.9%, respectively). For the high frequency of these concomitant conditions, a recently published Endocrine Society’s guideline recommended the use of scheduled insulin therapy instead of noninsulin therapies for glycemic management in hospitalized subjects with diabetes [40]. According to this recommendation, we observed that the prescriptions of insulin therapy increased significantly during the hospitalization of patients with diabetes in internal medicine and geriatric non-ICU wards. Notably, a sliding scale insulin regimen was prescribed to 19.6% of the patients. This regimen consisting in administration of short- or rapid-acting insulin 4 to 6 times a day, based on regularly obtained capillary blood glucose levels without concurrent use of basal or long-acting insulin, was not recommended by the 2019 Beers Criteria [12]. However, the most recent Endocrine Society Guidelines suggest both sliding scale and scheduled insulin regimens considering the lower risk of hypoglycemic events, but with a slightly higher daily plasma glucose levels and higher length of hospital stay observed in sliding scale insulin regimen as compared with scheduled insulin therapy [40]. Additionally, we found that almost half of patients with diabetes were discharged with insulin therapy, whereas there was a significant reduction in the prescription of noninsulin therapies at discharge as compared with the admission. The Endocrine Society Clinical Practice Guideline suggests that it may be reasonable to begin other noninsulin therapies, such as DPP-4 inhibitors, in stable patients prior to discharge as a part of a coordinated transition plan [40].

To the best of our knowledge, this is the first study that also evaluated the impact of appropriateness of anti-diabetes drugs in hospitalized elderly patients on mortality post-discharge. Indeed, we found that patients with type 2 diabetes not appropriately treated exhibited a 1.84-fold increased risk of mortality at 3 months as compared to patients appropriately treated. This increased risk remained significant also in adjusted model including age, sex and comorbidity index. In particular, the variables significantly associated with an increased risk of mortality at 3 months after discharge were age, CIRS and men. These results highlighting the importance of the appropriateness and the adherence to safety recommendations in the prescriptions of anti-diabetes drugs especially in elderly patients with comorbidities who could be exposed to an increased risk of mortality with an inappropriate treatment.

In the present study we also observed a lower prevalence of dementia in patients with diabetes as compared with patients without diabetes, in contrast to previous studies [41]; this discrepancy could be due to an underestimation of the diagnosis of dementia in hospitalized patients. Indeed, at admission more patients than those with an established diagnosis of dementia had Overt Cognitive impairment evaluated by Short Blessed Test, with no difference between patients with and without diabetes.

It was surprising to observe that at hospital admission about 3% of patients with type 2 diabetes were treated with the new classes of anti-diabetes drugs, such as GLP-1 RA, DPP-4 inhibitors and SGLT2 inhibitors, despite their efficacy and safety profile even in the elderly people with type 2 diabetes. It is conceivable that some concerns about an increased risk of euglycemic ketoacidosis and acute kidney injury especially in the patients with acute illness during the treatment with SGLT2 inhibitors have influenced the therapeutic choice. However, treatment with GLP-1 RA and DPP-4 inhibitors in hospitalized patients has been associated with similar glycemic control and lower rates of hypoglycemia compared with insulin regimens [3, 16, 17]. Moreover, given that treatment with saxagliptin has been associated with increased risk hospitalization for heart failure [18], we cannot exclude that DPP-4 inhibitors are prescribed with caution in older diabetic patients with heart failure.

Indeed, a recent meta-analysis has shown that although insulin therapy remains the preferred approach for glycemic management in hospitalized patients, treatment with DPP-4 inhibitors may be appropriate in select patients with type 2 diabetes, including those with well-managed diabetes and those with established noninsulin-requiring diabetes nearing hospital discharge [42]. A possible explanation for the low use of the new classes of anti-diabetes drugs observed in our analysis may be related to the fact that the elderly patients admitted to the REPOSI registry were enrolled from 2010 up to 2019 when data of cardiovascular outcome trial were not fully accrued and translated into clinical practice guideline. Indeed, at hospital discharge, we observed a nominally significant increase in the prescription of GLP-1 RA. Moreover, we observed a trend towards a reduction in the prevalence of the prescriptions of sulfonylureas and repaglinide and an increased use of DPP-4 inhibitors and to a lesser extent of GLP-RA and SGLT2 inhibitors, in the years 2018–2019 compared to the years 2010–2011. Clearly, future analyses on elderly patients admitted to medical and geriatric non-ICU wards after 2019 will be needed to determine if there is a greater adherence to recent guidelines on diabetes management and care in the elderly patients.

The present study has some strengths and limitations that merit consideration. A main strength is represented by the multicenter design of the REPOSI register with a large number of internal medicine and geriatric non-ICU wards throughout Italy providing a representative and unselected sample of older in-patients with multiple and severe diseases.

Nevertheless, this study has also some limitations. First, in the frame of the REPOSI register there is no information about diabetes duration and duration of the prescribed therapy. Second, HbA1c, which is the better indicator of long-term glycemic control, is lacking. Third, in the REPOSI register there is no information about any hypoglycemic events during the hospitalization. Furthermore, we observed a discrepancy in the number of patients diagnosed with diabetes at discharge that increased with respect as compared with the number of patients with diabetes diagnosis at the time of hospital admission, likely due to newly diagnosed type 2 diabetes diagnosed during hospitalization. Moreover, in the REPOSI register is not evaluated the economic status. Otherwise, in Italy, this is not an influencing factor concerning the antidiabetic therapy choice. This thanks to the nature of the national health system, which guarantee to all diabetic people to get the best and desired medicaments with a full reimbursement independently by the cost of the therapy. Because in Italy the health care is entirely tax financed, the present results are not influenced by the level of economic status of the participants at odds with other countries where health care relies on user payment. Finally, REPOSI register enrolled only Italian older in-patients and the results may not be generalizable to other ethnic groups or different geographical areas.

## Conclusions

Overall, the present study shows a poor adherence to recent guidelines on diabetes management and care in hospitalized elderly people in internal medicine and geriatric non-ICU wards assessed from 2010 to 2019. Notably, we found a high proportion of inappropriate use of sulfonylureas according to the 2019 AGS Beers criteria. Furthermore, at hospital admission only ~ 3% of elderly patients with type 2 diabetes were treated with the new classes of anti-diabetes drugs, such as GLP-1 RA, DPP-4 inhibitors, and SGLT2 inhibitors, despite it has been shown to be effective, and safe drugs in elderly patients and we observed a trend towards an increase in their prescriptions in the years 2018–2019 compared to the years 2010–2011. Importantly, the inappropriateness prescriptive of the anti-diabetes drugs was associated with an increased risk of mortality at 3 months in elderly patients with type 2 diabetes hospitalized. These results highlighting the importance of the appropriateness and the adherence to safety recommendations in the prescriptions of anti-diabetes drugs especially in elderly patients with comorbidities. Future analyses on elderly patients admitted to medical and geriatric non-ICU wards after 2019 are needed to explore if there is a greater adherence to recent guidelines on diabetes management and care in elderly patients.

## Supplementary Information

Below is the link to the electronic supplementary material.

Supplementary file1 (DOCX 24 KB)

## Data Availability

The datasets used and analysed during the current study are available from the corresponding author on reasonable request.
