# Correction to: Prescription appropriateness of anti-diabetes drugs in elderly patients hospitalized in a clinical setting: evidence from the REPOSI Register

**DOI:** 10.1007/s11739-023-03361-1

**Published:** 2023-08-03

**Authors:** Elena Succurro, Alessio Novella, Alessandro Nobili, Federica Giofrè, Franco Arturi, Angela Sciacqua, Francesco Andreozzi, Antonello Pietrangelo, Giorgio Sesti, REPOSI Investigators, Francesco Perticone, Francesco Violi, Salvatore Corrao, Alessandra Marengoni, Mauro Tettamanti, Luca Pasina, Carlotta Franchi, Carlotta Franchi, Mauro Tettamanti, Gabriella Miglio, Mauro Tettamanti, Ilaria Ardoino, Silvia Cantiero, Domenico Prisco, Elena Silvestri, Giacomo Emmi, Alessandra Bettiol, Irene Mattioli, Matteo Mazzetti, Gianni Biolo, Michela Zanetti, Giacomo Bartelloni, Michele Zaccari, Massimiliano Chiuch, Ilaria Martini, Matteo Pirro, Graziana Lupattelli, Vanessa Bianconi, Riccardo Alcidi, Alessia Giotta, Massimo R Mannarino, Domenico Girelli, Fabiana Busti, Giacomo Marchi, Mario Barbagallo, Ligia Dominguez, Vincenza Beneduce, Federica Cacioppo, Salvatore Corrao, Giuseppe Natoli, Salvatore Mularo, Massimo Raspanti, Christiano Argano, Federica Cavallaro, Marco Zoli, Giuseppe Orio, Eleonora Magnolfi, Giovanni Serafini, Mattia Brunori, Ilaria Lazzari, Angelo Simili, Giovanna Fabio, Margherita Migone De Amicis, Giacomo Luca, Natalia Scaramellini, Valeria Stefano, Simona Leoni, Sonia Seghezzi, Alessandra Danuto Di Mauro, Diletta Maira, Marta Mancarella, Tiziano Lucchi, Marta Clerici, Simona Leoni, Giulia Bonini, Federica Conti, Silvia Prolo, Maddalena Fabrizi, Miriana Martelengo, Giulia Vigani, Paola Nicolini, Antonio Sabatino, Emanuela Miceli, Martina Pisati, Lavinia Pitotti, Valentina Antoci, Ginevra Cambiè, Lavinia Pitotti, Valentina Antoci, Roberto Pontremoli, Valentina Beccati, Giulia Nobili, Giovanna Leoncini, Jacopo Alberto, Federico Cattaneo, Luigi Anastasio, Lucia Sofia, Maria Carbone, Francesco Cipollone, Ilaria Rossi, Emanuele Valeriani, Damiano D’Ardes, Alessia Cipollone, Lucia Esposito, Simona Sestili, Ermanno Angelucci, Gerardo Mancuso, Daniela Calipari, Mosè Bartone, Roberto Manetti, Marta Sircana, Maria Berria, Alessandro Delitala, Maurizio Muscaritoli, Alessio Molfino, Enrico Petrillo, Antonella Giorgi, Christian Gracin, Giovanni Imbimbo, Giuseppe Romanelli, Alessandra Marengoni, Andrea Volpini, Daniela Lucente, Francesca Manzoni, Annalisa Pirozzi, Alberto Zucchelli, Thelma Geneletti, Antonio Picardi, Giuseppe Bellelli, Maurizio Corsi, Cesare Antonucci, Chiara Sidoli, Giulia Principato, Alessandra Bonfanti, Hajnalka Szabo, Paolo Mazzola, Andrea Piazzoli, Maurizio Corsi, Bruno Tassone, Antonio Brucato Teresa De Falco, Enrica Negro, Martino Brenna, Lucia Trotta, Fabrizio Fabris, Irene Bertozzi, Giulia Bogoni, Tancredi Prandini, Francesco Ratti, Chiara Zurlo, Lorenzo Cerruti, Elisabetta Cosi, Elisa Reni, Roberto Manfredini, Benedetta Boari, Alfredo Giorgi, Ruana Tiseo, Caterina Savriè, Fabio Fabbian, Giuseppe Paolisso, Claudia Catalano, Irene Meo, Carlo Sabbà, Patrizia Suppressa, Giovanni Michele De Vincenzo, Alessio Comitangelo, Emanuele Amoruso, Carlo Custodero, Giuseppe Re, Ivano Barnaba, Andrea Schilardi, Luigi Fenoglio, Andrea Falcetta, Salvatore D’Aniano, Silvia Tiraboschi, Annalisa Cespiati, Giovanna Oberti, Giordano Sigon, Felice Cinque, Lucia Colavolpe, Jaqueline Currà, Francesca Alletto, Natalia Scaramellini, Simona Leoni, Alessandra Danuta Di Mauro, Gianpaolo Benzoni, Flora Peyvandi, Raffaella Rossio, Giulia Colombo, Pasquale Agosti, Erica Pagliaro, Eleonora Semproni, Ciro Canetta, Valter Monzani, Valeria Savojardo, Giuliana Ceriani, Christian Folli, Tiziana Tognin, Francesco Purrello, Antonino Pino, Salvatore Piro, Renzo Rozzini, Lina Falanga, Stefano Boffelli, Camillo Ferrandina, Francesca Mazzeo, Elena Spazzini, Giulia Cono, Giulia Cesaroni, Francesco Violi, Ludovica Perri, Luigina Guasti, Francesca Rotunno, Luana Castiglioni, Andrea Maresca, Alessandro Squizzato, Leonardo Campiotti, Alessandra Grossi, Francesco Dentali, Veronica Behnke, Maria Perticone, Raffaele Maio, Aleandra Scozzafava, Valentino Condoleo, Elvira Clausi, Giuseppe Armentaro, Alberto Panza, Valentino Condoleo, Vincenzo Stanghellini, Eugenio Ruggeri, Sara Vecchio, Ilaria Benzoni, Salvatore Minisola, Luciano Colangelo, Mirella Cilli, Giancarlo Labbadia, Jessica Pepe, Pietro Castellino, Luca Zanoli, Agostino Gaudio, Anastasia Xourafa, Concetta Spichetti, Serena Torre, Alfio Gennaro, Alberto Ballestrero, Fabio Ferrando, Roberta Gonella, Domenico Cerminara, Paolo Setti, Chiara Traversa, Camilla Scarsi, Giuseppe Famularo, Patrizia Tarsitani, Tiziana Morretti, Andrea Aglitti, Stefano Giacco, Davide Firinu, Giulia Costanzo, Salvatore Chessa, Giuseppe Montalto, Anna Licata, Angelo Rizzo, Francesco Corica, Giorgio Basile, Antonino Catalano, Federica Bellone, Concetto Principato, Angelo Cocuzza, Patrizia Mecocci, Carmelinda Ruggiero, Virginia Boccardi, Tiziana Meschi, Andrea Ticinesi, Antonio Nouvenne, Mario Pirisi, Daniele Sola, Mattia Bellan, Roberto Quadri, Erica Larovere, Marco Novelli, Emilio Simeone, Rosa Scurti, Fabio Tolloso, Roberto Tarquini, Alice Valoriani, Silvia Dolenti, Giulia Vannini, Riccardo Volpi, Pietro Bocchi, Alessandro Vignali, Sergio Harari, Chiara Lonati, Federico Napoli, Italia Aiello, Teresa Salvatore, Lucio Monaco, Carmen Ricozzi, Francesca Coviello, Christian Catalini, Alberto Pilotto, Ilaria Indiano, Federica Gandolfo, Davide Gonella, Ranuccio Nuti, Roberto Valenti, Martina Ruvio, Silvia Cappelli, Alberto Palazzuoli, Vittorio Durante, Daniela Tirotta, Giovanna Eusebi, Moreno Tresoldi, Enrica Bozzolo, Sarah Damanti, Massimo Porta, Miriam Gino, Bianca Pari, Edoardo Pace

**Affiliations:** 1 Department of Medical and Surgical Sciences, University Magna Graecia of Catanzaro, Viale Europa, 88100 Catanzaro, Italy; 2 Department of Health Policy, Istituto Di Ricerche Farmacologiche Mario Negri IRCCS, 20156 Milan, Italy; 3 Division of Internal Medicine 2nd Center for Haemochromatosis, University Hospital of Modena, 41124 Modena, Italy; 4 Department of Clinical and Molecular Medicine, University of Rome-Sapienza, 00189 Rome, Italy

**Correction to: Internal and Emergency Medicine** 10.1007/s11739-023-03254-3

In this article the names of a few collaborators and some data in Table 5 were missing. It has been corrected.

The original article has been corrected.

