# Supplementary material for: Prescription appropriateness of anti-diabetes drugs in elderly patients hospitalized in a clinical setting: evidence from the REPOSI Register
Source: Intern Emerg Med. 2023 Mar 25;18(4):1049–63. doi: 10.1007/s11739-023-03254-3 (PMC10326139; doi:10.1007/s11739-023-03254-3)
Supplement: Supplementary file 1 — Supplementary file1 (DOCX 24 KB) [file 11739_2023_3254_MOESM1_ESM.docx]

**Table S1 Prevalence of prescriptions of glucose-lowering drugs stratified by years of enrollment of elderly patients with type 2 diabetes patients in the REPOSI register, at hospital admission and discharge**

| **Admission** | **2010-2011**  **N=240** | **2012-2013**  **N=348** | **2014-2015**  **N=360** | **2016-2017**  **N=348** | **2018-2019**  **N=328** |
| --- | --- | --- | --- | --- | --- |
| Metformin | 95 (39.6) | 122 (35.1) | 138 (38.3) | 129 (37.1) | 128 (39.0) |
| Pioglitazone | 2 (0.8) | 4 (1.2) | 5 (1.4) | 5 (1.4) | 7 (2.1) |
| Sulfonylureas | 63 (26.3) | 65 (18.7) | 60 (16.7) | 45 (12.9) | 34 (10.4) |
| SGLT2 inhibitors | 0 | 0 | 0 | 0 | 3 (0.9) |
| DPP-IV inhibitors | 0 | 8 (2.3) | 8 (2.2) | 11 (3.2) | 18 (5.5) |
| GLP-1 RA | 0 | 0 | 0 | 0 | 2 (0.6) |
| Insulin therapy | 87 (36.3) | 115 (33.1) | 150 (41.7) | 140 (40.2) | 113 (34.5) |
| Acarbose | 2 (0.8) | 6 (1.7) | 9 (2.5) | 13 (3.7) | 10 (3.1) |
| Repaglinide | 30 (12.5) | 53 (15.2) | 43 (11.9) | 37 (10.6) | 22 (6.7) |
|  |  |  |  |  |  |
| **Discharge** | **2010-2011**  **N=252** | **2012-2013**  **N=368** | **2014-2015**  **N=370** | **2016-2017**  **N=351** | **2018-2019**  **N=355** |
| Metformin | 82 (32.5) | 100 (27.2) | 95 (25.7) | 91 (25.9) | 104 (29.3) |
| Pioglitazone | 0 | 2 (0.5) | 1 (0.3) | 0 | 4 (1.1) |
| Sulfonylureas | 41 (16.3) | 37 (10.1) | 38 (10.3) | 20 (5.7) | 16 (4.5) |
| SGLT2 inhibitors | 0 | 0 | 0 | 0 | 4 (1.1) |
| DPP-IV inhibitors | 3 (1.2) | 8 (2.2) | 4 (1.1) | 10 (2.9) | 21 (5.9) |
| GLP-1 RA | 0 | 0 | 1 (0.3) | 1 (0.3) | 4 (1.1) |
| Insulin therapy | 115 (45.6) | 146 (39.7) | 180 (48.7) | 169 (48.2) | 149 (42.0) |
| Acarbose | 0 | 4 (1.1) | 5 (1.4) | 7 (2.0) | 8 (2.3) |
| Repaglinide | 33 (13.1) | 65 (17.7) | 38 (10.3) | 21 (6.0) | 17 (4.8) |

**Table S2 Prevalence of prescriptions of glucose-lowering drugs stratified by by geographic areas of centers participating to REPOSI register that enrolled the patients (Northern, Central and Southern Italy), at hospital admission and discharge**

| **At Admission**  **(14 missing)** | Northern Italy  N=812 | Central Italy  N=265 | Southern Italy  N=533 | P-value |
| --- | --- | --- | --- | --- |
| Metformin  Inappropriately treated | 300 (37.0)  48 (16.0) | 101 (38.1)  19 (18.8) | 206 (38.7)  26 (12.6) | 0.81  0.33 |
| Pioglitazone  Inappropriately treated | 11 (1.4)  0 | 4 (1.5)  0 | 7 (1.3)  0 | 0.97  - |
| Sulfonylureas  Inappropriately treated | 153 (18.8)  41 (26.8) | 51 (19.2)  15 (29.4) | 61 (11.4)  20 (32.8) | 0.0007  0.68 |
| SGLT2 inhibitors  Inappropriately treated | 0  - | 0  - | 3 (0.6)  0 | 0.05  - |
| DPP-IV inhibitors  Inappropriately treated | 24 (3.0)  0 | 8 (3.0)  0 | 12 (2.3)  0 | 0.71  - |
| GLP-1 RA  Inappropriately treated | 0  - | 0  - | 1 (0.2)  0 | 0.36  - |
| Insulin therapy  Inappropriately treated | 277 (34.1)  54 (19.5) | 98 (37.0)  21 (21.4) | 226 (42.4)  43 (19.0) | 0.0088  0.88 |
| Acarbose  Inappropriately treated | 20 (2.5)  0 | 3 (1.1)  0 | 16 (3.0)  0 | 0.27  - |
| Repaglinide  Inappropriately treated | 95 (11.7)  0 | 27 (10.2)  1 (3.7) | 62 (11.6)  1 (1.6) | 0.78  0.23 |
| Inappropriately treated | 137 (16.9) | 56 (21.1) | 89 (16.7) | 0.24 |
| **At discharge**  (17 Missing) | Northern Italy  N=839 | Central Italy  N=276 | Southern Italy  N=564 | P-value |
| Metformin  Inappropriately treated | 224 (26.7)  27 (12.1) | 81 (29.4)  9 (11.1) | 162 (28.7)  11 (6.8) | 0.58  0.22 |
| Pioglitazone  Inappropriately treated | 3 (0.4)  0 | 1 (0.4)  0 | 2 (0.4)  0 | 0.99  - |
| Sulfonylureas  Inappropriately treated | 94 (11.2)  26 (27.7) | 31 (11.2)  9 (29.0) | 26 (4.6)  9 (34.6) | <0.0001  0.79 |
| SGLT2 inhibitors  Inappropriately treated | 0  - | 0  - | 4 (0.7)  0 | 0.019  - |
| DPP-IV inhibitors  Inappropriately treated | 17 (2.0)  0 | 8 (2.9)  0 | 19 (3.4)  0 | 0.29  - |
| GLP-1 RA  Inappropriately treated | 0  - | 0  - | 5 (0.9)  2 | 0.007  - |
| Insulin therapy  Inappropriately treated | 340 (40.5)  77 (22.7) | 131 (47.5)  23 (17.6) | 280 (49.7)  47 (16.8) | 0.0021  0.15 |
| Acarbose  Inappropriately treated | 13 (1.6)  0 | 2 (0.7)  0 | 9 (1.6)  0 | 0.56  - |
| Repaglinide  Inappropriately treated | 91 (10.9)  0 | 26 (9.4)  0 | 56 (9.9)  0 | 0.75  - |
| Inappropriately treated | 128 (15.3) | 41 (14.9) | 67 (11.9) | 0.19 |

**Table S3 Prevalence of inappropriateness causes for antidiabetic drugs according to prescriptive and BEERS criteria among not-appropriately treated subjects, at hospital admission and discharge**

|  | **At admission** | **At discharge** |
| --- | --- | --- |
| *Inappropriate Metformin* | | |
| eGFR <30 | 51 (54.3) | 14 (29.2) |
| Acute Myocardial Infarction | 20 (21.3) | 19 (39.6) |
| Respiratory Failure | 19 (20.2) | 12 (25.0) |
| Acute Illness | 13 (13.8) | 11 (22.9) |
| Metabolic Acidosis | 0 | 1 (2.1) |
| *Inappropriate Sulfonylureas* | | |
| eGFR <15 | 7 (100) | 0 |
| Kidney Failure | 0 | 1 (100) |
| *Inappropriate GLP-1 RA* | | |
| eGFR <30 | 0 | 2 (100) |
| *Inappropriate Repaglinide* | | |
| Pancreatitis | 1 (50.0) | 0 |
| Liver Failure | 1 (50.0) | 0 |
| **BEERS criteria** | | |
| *Inappropriate Sulfonylureas* | | |
| Sulfonylureas, long acting -  Chlorpropamide, Glimepiride, Glyburide (also known as glibenclamide) | 76 (28.5) | 44 (29.0) |
| *Inappropriate Insulin therapy* | | |
| sliding scale (insulin regimens  containing only short- or rapid-acting insulin  dosed according to current blood glucose  levels without concurrent use of basal or  long-acting insulin) | 119 (19.7) | 149 (19.6) |
